# Next-generation sequencing revealed recurrent *ZFPM1* mutations in encapsulated papillary carcinoma of the breast

**DOI:** 10.1038/s41698-021-00180-5

**Published:** 2021-05-18

**Authors:** Xuguang Liu, Xin Huang, Yan Bai, Zhiwen Zhang, Tiefeng Jin, Huanwen Wu, Zhiyong Liang

**Affiliations:** 1 Department of Pathology, Complex Severe and Rare Disease, Molecular Pathology Research Center, Peking Union Medical College Hospital, Chinese Academy of Medical Sciences and Peking Union Medical College, Beijing, China; 2 Department of Pathology, Tongji Hospital, Tongji Medical College, Huazhong University of Science and Technology, Wuhan, China; 3 Department of Breast Surgery, Peking Union Medical College Hospital, Beijing, China; 4 Department of Pathology and Cancer Research Center, Yanbian University Medical College, Yanji, China

**Keywords:** Oncogenesis, Breast cancer

## Abstract

Encapsulated papillary carcinoma (EPC) of the breast is a rare subtype of tumor. To date, the genetic abnormalities underlying EPC remain elusive. The purpose of this study was to gain further insight into EPC mutation profile. Forty-one EPCs diagnosed from 2015 to 2018 were included. Twenty-six EPCs were submitted to whole-exome sequencing (WES), and a 185 gene-targeted sequencing panel was designed to validate the results of the 26 EPCs that underwent WES and 15 additional cases. Recurrently mutated genes were further confirmed by Sanger sequencing. Our study revealed multiple recurrently mutated genes including PI3K-AKT-mTOR pathway genes (*PIK3CA*, *AKT1*, *ULK1*, *MAP3K1, MAP2K4*, *RHOA*, and *PTEN*) (27/41, 65.8%) and chromatin modification genes (*ZFPM1*, *GATA3*, *CTCF*, and *KMT2C*) (21/41, 51.2%) in EPC. Importantly, somatic *ZFPM1* mutations existed in 9/41 (21.9%) of the EPCs. The frequency of *ZFPM1* mutations in the EPCs was significantly higher than that of other tumor types. Of the nine *ZFPM1* mutations, seven were frameshift mutations, and the remaining two were nonsense mutations. Moreover, a significant concurrence of *ZFPM1* and PI3K-AKT-mTOR mutations were revealed in the EPCs. Of note, no *TP53* mutations were detected in our EPCs, whereas it was detected in a considerable proportion of the luminal A invasive ductal carcinomas of no special type (IDC-NSTs) from TCGA. We reveal that recurrent somatic *ZFPM1* mutation is characteristic of EPC and concurred with mutations in the PI3K-AKT-mTOR pathway. The distinctive genetic features of EPC might underlie its special histological structures and indolent behavior.

## Introduction

Encapsulated papillary carcinoma (EPC) is a rare subtype of breast cancer. EPC can be categorized as either a special invasive carcinoma, based on a lack of peripheral myoepithelial cells in the fibrous tumor capsule (as determined by immunohistochemical staining of p63, SMA, etc.), or as a form of ductal carcinoma in situ, due to its indolent biological behavior and excellent prognosis^1–3^. Some pathologists consider EPC as part of a spectrum of progression from in situ to invasive carcinoma^4^. Although some studies have compared EPC with in situ and invasive carcinomas at the genomic level, the results have been contradictory^2,5^.

Large-scale genomic technologies, such as whole-exome sequencing (WES), have been successfully applied to identify many genetic alterations in breast invasive ductal carcinomas of no special type (IDC-NST), leading to great progress in the characterization of its mutational landscape^6,7^. In addition, specific genetic mutations have been identified in specific breast cancers, such as the *IDH2* hotspot mutation (R172) in tall cell carcinoma with reverse polarity of the breast and the *CDH1* truncating mutations in invasive lobular carcinoma^8,9^. Transcriptomic analyses have demonstrated that the expression of cell migration genes in EPC is reduced compared with other types of papillary carcinoma^5^. Furthermore, some genetic alterations have been identified in EPCs from RNA-sequencing data, including mutations in *PIK3CA* (H1047R) and *AKT1* (L52H)^5,10^. However, the genetic abnormalities underlying EPC have yet to be well clarified. One of the major reasons for this is the small sample size available in previous studies due to its rarity.

In this study, WES and targeted sequencing (TS) were performed in a relatively large cohort of EPCs to explore the mutational profile and reveal characteristic molecular genetic alterations in EPC.

## Results

### Clinical features of the study population

**Fig. 1:**
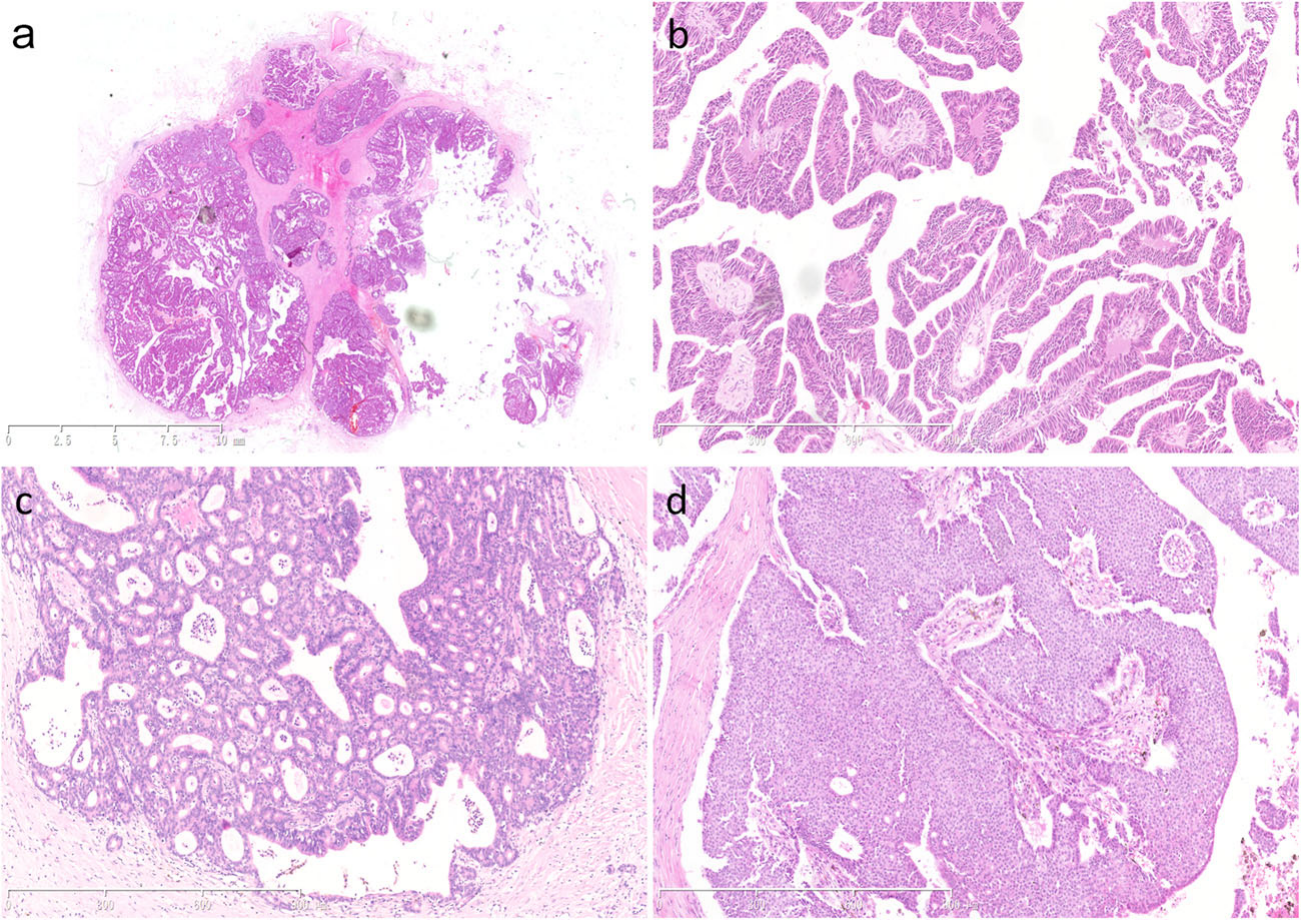
Histopathological features of EPCs. **a** A scan of an EPC in a low power field (4×). **b** Papillary structure (100×, H&E staining). **c** Cribriform structure (100×, H&E staining). **d** Solid structure (100×, H&E staining).

All 41 patients were female with a median age of 60.5 years (range: 30–83 years). None of the patients received neoadjuvant therapy. All patients in this cohort were diagnosed with low- to intermediate-grade EPCs. We observed that 60.9% (25/41) of cases were pure EPC, and 39.1% (16/41) of cases were EPC with invasive carcinoma (EPC-IC). In EPC-IC, the majority of accompanied invasive carcinoma were IDC-NST (Grade 1), and mucinous carcinoma was found in only one case. In terms of predominant architecture in EPC, papillary and cribriform structure exhibited in 92.7% (38/41) of cases, whereas solid structure was found in 7.3% (3/41) (Fig. 1). 80.5% (33/41) of patients were available for assessment of regional lymph node status. Only one patient had sentinel lymph node micrometastasis (<1 mm), and the remaining patients did not present lymph node metastasis. Our EPCs were all high ER- and PR- positive but Her-2- negative. The average Ki-67 index was 7.3% (range: 1–25%). Most of EPC could be classified as “luminal-A like” according to St. Gallen International Expert Consensus^11^. Follow-up data were available for 40 patients. The median follow-up was 36.5 months (range: 5–62 months). There were no local recurrences, distant metastases, or cancer-related deaths during the follow-up period (Supplementary Data 1). The detailed clinicopathological features were listed in Supplementary Data 1.

### Somatic mutational landscape of EPCs

WES was performed on paired tumor and germline DNA from 26 patients. All tumor DNA was extracted from fixed paraffin-embedded (FFPE) tissues. Of the 26 patients, germline DNA was extracted from whole-blood in 16 patients and FFPE normal tissues in the remaining 10 patients.

**Fig. 2:**
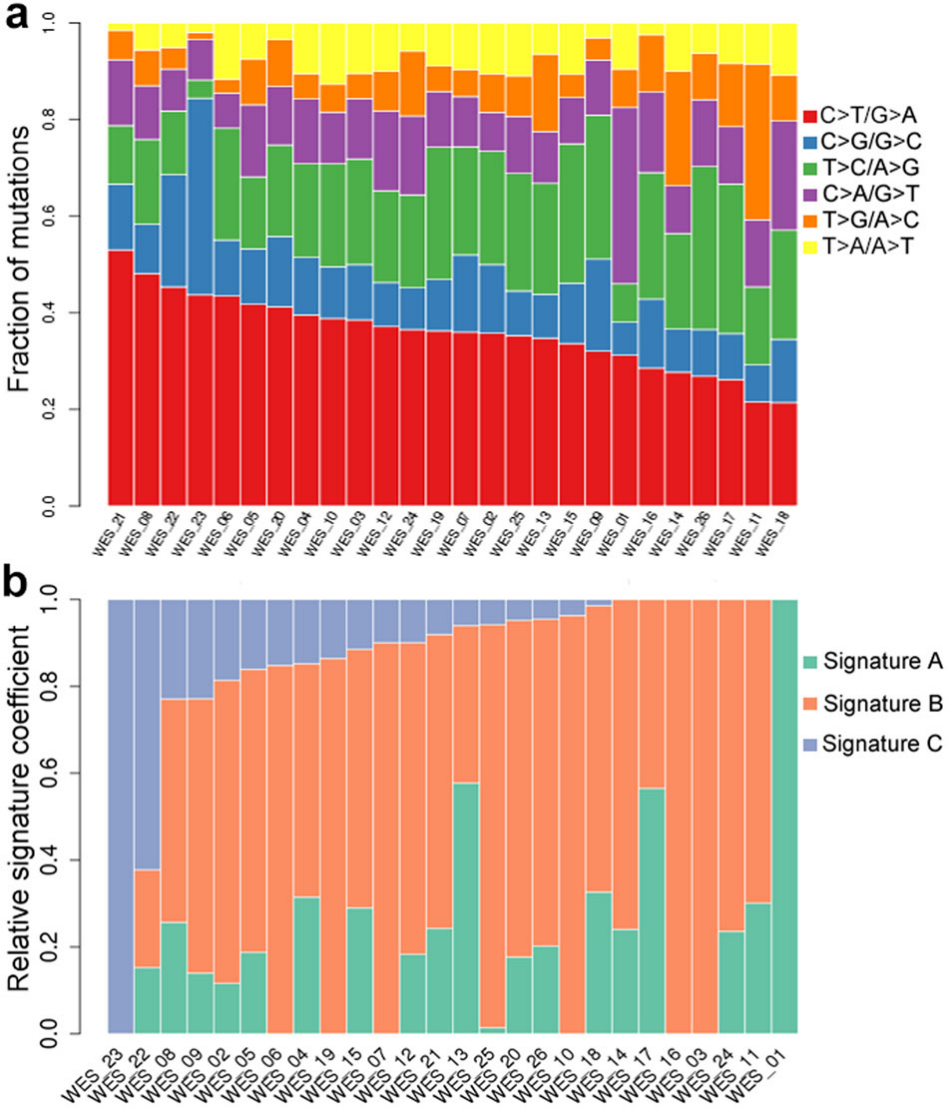
Mutation spectra and mutation signatures among EPC samples. **a** The left column depicted the mutation spectrum across all tumor samples. Red represented C > T/G > A mutations, blue represents C > G/G > C mutations, green represented T > C/A > G mutations, purple represented C > A/G > T mutations, orange represented T > G/A > C mutations, and yellow represented T > A/A > T mutations. The Y-axis indicated the proportion of samples with the mutations. **b** Nonnegative matrix factorization cluster analyses acquired three different mutation signatures. The Y-axis indicated the proportion of acquired mutation signatures.

The somatic mutations were listed in Supplementary Data 2. An average of 40 Gb data was generated from each sample. The average depths of targeted exome regions in tumors and matched blood/normal tissues were ×400 (range 342–616) and ×100 (range 88–166), respectively. More than 98.79% of the targeted regions were covered sufficiently for confident variant calling (≥10× depth). For nucleotide substitutions, the predominant types of SNVs in EPCs were C > T/G > A and T > C/A > G transitions and C > A/G > T transversions. Three signatures (A, B, and C) were revealed in EPCs by nonnegative matrix factorization clustering^12^. Signature B showed the largest contribution, which was found to be similar to Signature 5 (*α* = 0.83). This signature was featured with T > C substitutions and common in all cancer types. Signature C was similar to Signature 13 (*α* = 0.83), which was associated to old age and common in invasive breast cancer^7^. In this cohort, 80% of EPC patients were postmenopausal females (Age > 50). This signature could be in part explained by the age of EPC. Lastly, Signature A was associated with Signature 29 (*α* = 0.66). The underlying molecular mechanisms of signature A are transcriptional strand bias for C > A mutations and common in individuals with a tobacco chewing habit, while the association between signature A and EPC was unknown (Fig. 2).

Recurrent mutated genes including *PIK3CA* (13/26 50.0%), *ZFPM1* (6/26, 23.0%), *GATA3* (4/26, 15.3%), *AKT1* (2/26, 7.7%) and *CTCF* (2/26, 7.7%) were identified by WES (Supplementary Data 2). Among these recurrent mutated genes, *ZFPM1* gene mutations have not been reported in breast cancer.

**Fig. 3:**
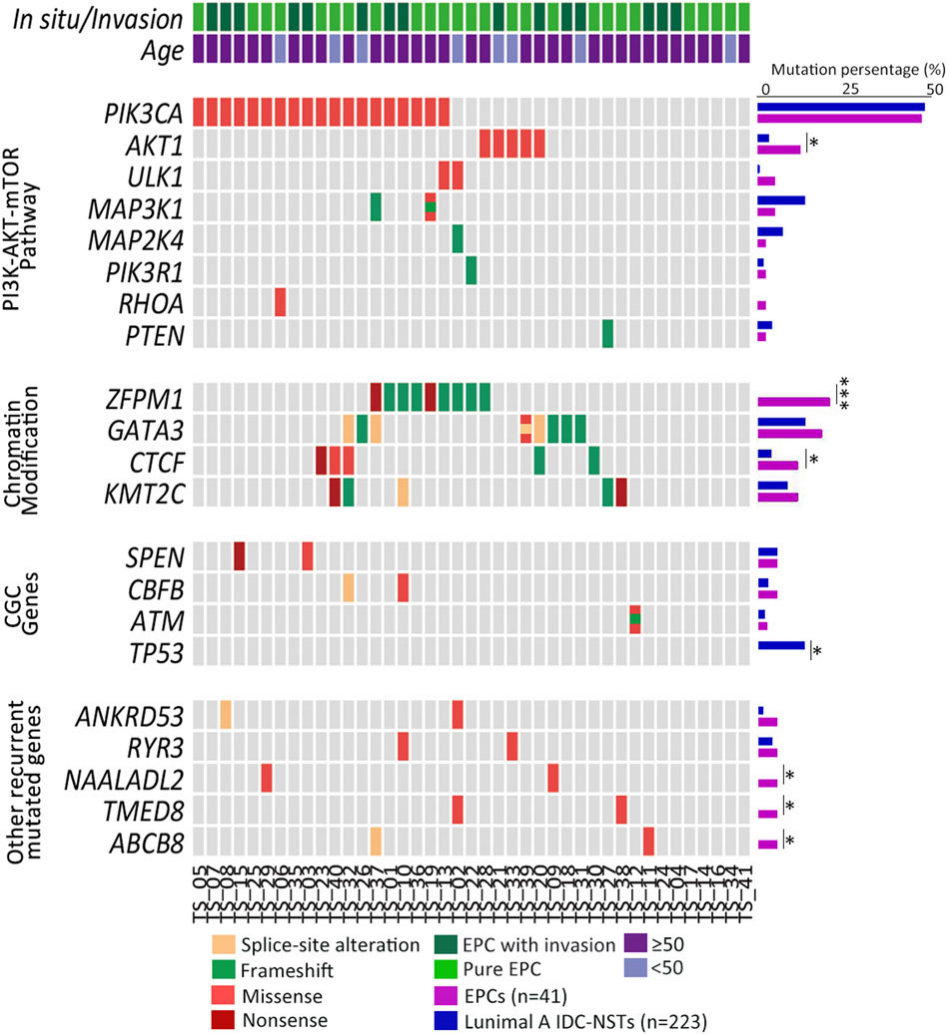
Somatic mutation genes in 41 pairs of EPC. Each column denoted an individual tumor, and each row represented a gene. The percent of mutated cases in this study (purple bars) and in the luminal A IDC-NST data from TCGA (blue bars) was shown. Two-tailed Chi-square and Fisher’s exact test, *p < 0.05, ***p < 0.001. IDC-NST invasive carcinoma of no special type, CGC Cancer Gene Census.

Deep TS (185 gene panel) was performed in 41 paired EPCs (26 WES cases and 15 additional cases). Paired tumor and normal FFPE tissues were used for the additional 15 patients. The mean coverage of the targeted genes was 1137× (range 474–1830) in tumor tissues and 517× (range 40–1356) in paired normal tissues. By TS, 179 somatic mutations, including 27 small coding insertions/deletions (indels), 10 nonsense mutations, 134 missense mutations, and 8 splice-site changes were detected in 41 EPC cases. Of 134 missense mutations, 89 were predicted to have a high probability of pathogenicity by polymorphism phenotyping (PolyPhen) and SIFT algorithm (Supplementary Data 3). We identified multiple frequently somatic mutated genes in EPCs and divided them into several subsets including PI3K-AKT-mTOR pathway genes (*PIK3CA*, *AKT1*, *ULK1*, *MAP3K1, MAP2K4*, *RHOA*, and *PTEN*) (27/41, 65.8%), chromatin modification genes (*ZFPM1*, *GATA3*, *CTCF*, and *KMT2C*) (21/41, 51.2%), and Cancer Gene Census genes (CGC) (*SPEN*, *CBFB*, and *ATM*) (Fig. 3). Recurrent genes mutations were confirmed by Sanger sequencing (Supplementary Fig. 1).

### Characteristics of somatic *ZFPM1* mutations in EPCs

**Fig. 4:**
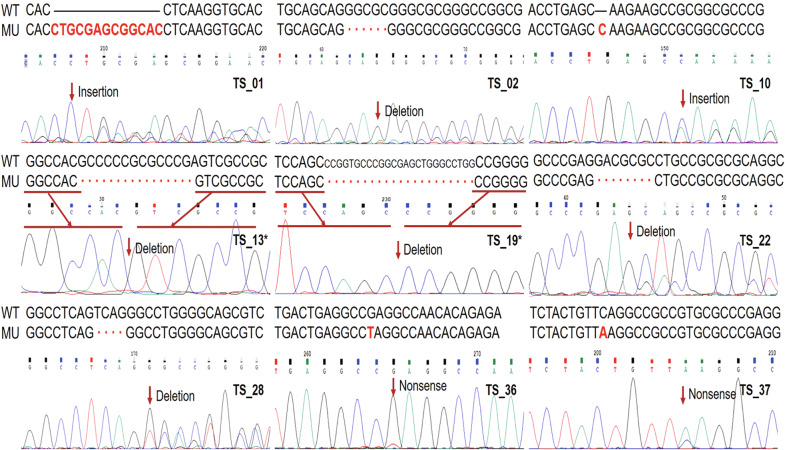
Somatic *ZFPM1* mutations were confirmed in nine EPCs by Sanger sequencing. Top: DNA sequences for wild-type and mutated alleles. Below: Sanger sequencing validation electrophoretograms. WT, wild-type allele; MU mutated allele. * A further explanation for TS_13&19 in Supplementary Fig. 4.

Somatic *ZFPM1* mutations were identified by WES in six cases (6/26, 23.0%), and an additional 3 tumor samples with *ZFPM1* mutations were revealed in 15 additional cases by TS. Ultimately, somatic *ZFPM1* mutations existed in nine EPCs (21.9%, 9/41). All mutations were confirmed by Sanger sequencing (Fig. 4 and Supplementary Fig. 4).

**Fig. 5:**
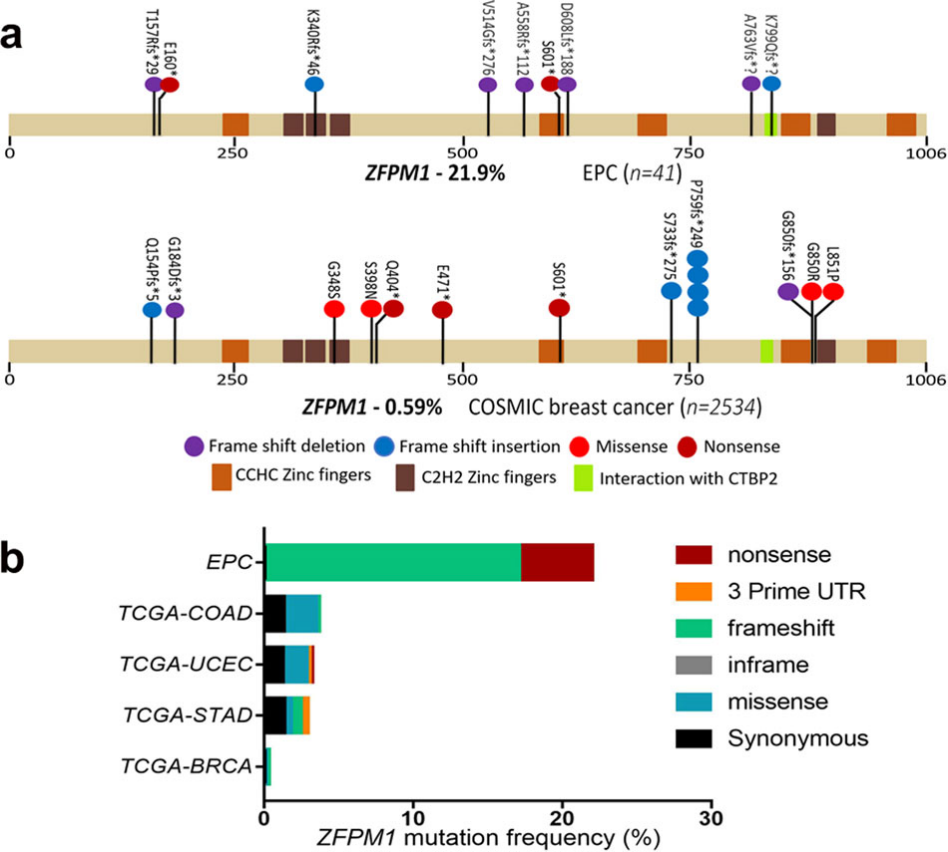
Mapping of mutation sites in *ZFPM1* from our series and the COSMIC dataset. **a** The functional domains of the altered proteins were identified based on the UniProt database. The upper bar depicted *ZFPM1* mutations in EPCs by TS (*n* = 41). The bar below showed *ZFPM1* mutations in breast cancers from the COSMIC dataset. **b** *ZFPM1* mutation frequency in EPCs compared to that in several TCGA projects. COAD, colon adenocarcinoma (17/461, 3.68%); STAD, stomach adenocarcinoma (13/447, 2.90%); UCEC, uterine corpus endometrial carcinoma (18/560, 3.21%); BRCA, invasive breast carcinoma (3/986, 0.30%).

The human *ZFPM1* gene lies on chromosome 16q24 and encodes a nine-zinc-finger protein (1006 aa) that is highly homologous to murine Fog1, which is an essential cofactor that acts via the formation of a heterodimer with transcription factors of the GATA family: GATA1, GATA2, and GATA3. ZFPM1 is also known as the Friend of GATA-1 (FOG1). In our series, most of *ZFPM1* mutations located in exon 5 or exon 10. Of the nine *ZFPM1* mutations, 77.8% (7/9) were frameshift mutations, five of which were predicted to cause protein truncation, and two of which were predicted to extend the C-terminus. The remaining two cases were nonsense mutations resulting in an early stop codon (Fig. 5a). As a result, all nine *ZFPM1* mutations were predicted to fail in encoding natural C-terminal zinc-finger motifs.

To date, *ZFPM1* mutations have not been described in breast cancer studies. In COSMIC database (tissue: breast; *n* = 2534), fifteen (0.59%, 15/2534) invasive breast carcinomas were found with *ZFPM1* mutations. Of the fifteen cases with mutations, the majority were breast IDC-NSTs with ER- and PR-positive immunophenotype, with one case exhibiting papillary architecture histologically. The frequency of *ZFPM1* mutations in our EPCs was also significantly higher than that in other tumor types according to the TCGA database, including colon adenocarcinoma, uterine corpus endometrial carcinoma, stomach adenocarcinoma, and invasive breast carcinoma (*p* < 0.05, Chi-square test; Fig. 5b, Supplementary Data 4). All *ZFPM1* mutations identified in EPCs were novel, except for S601* in the TCGA database. These results indicate that recurrent somatic *ZFPM1* mutation is characteristic of EPC. Interestingly, recurrent *ZFPM1* mutations in EPCs co-occurred with mutations affecting canonical PI3K-AKT-mTOR pathway genes (*p* = 0.017 for co-occurrence, Fisher’s exact test; Supplementary Fig. 3). Six *ZFPM*-mutated cases accompanied with *PIK3CA* hotspot mutations (including two H1047R, three E545K, and one N345K). The remaining three cases harbored concurring *ALK1* (E17K), *PIK3R1* (Y165fs/G35fs) and *ULK1* (H72Q) mutation, respectively.

**Fig. 6:**
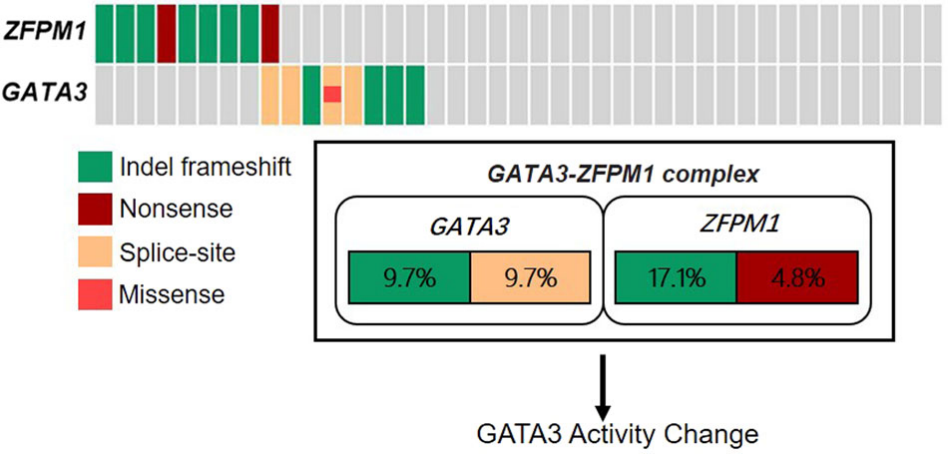
The mutations of *ZFPM1* and *GATA3*. Mutual exclusivity analysis between *ZFPM1* and *GATA3* mutations in EPC. The frequency of alteration in EPCs was shown in the box, *p* = 0.659, Fisher’s exact test. Each column denotes an individual tumor, and each row represents a mutation. The different colors represented different mutation types.

ZFPM proteins are tightly associated with GATA functions^13–17^. ZFPM1 plays a vital role in binding with GATA proteins and the function of GATA3 could be changed by the *ZFPM1* mutation^16–18^. We found that mutations of *GATA3* and *ZFPM1* seemed to show a mutually exclusive pattern in our EPCs, although there was no statistically significant (*p* = 0.659 for mutual exclusivity, Fisher’s exact test; Fig. 6).

### Mutations affecting the PI3K-AKT-mTOR pathway in EPCs

In total, 65.8% (27/41) of EPCs harbored at least one somatic nonsynonymous mutation in the PI3K-AKT-mTOR signaling pathway. Nineteen cases (46.3%, 19/41) harbored *PIK3CA* hotspot mutations, including nine H1047R/L, six E545K, one Q546K, one E542K, one N345K, and one G118D. Activating mutations in *AKT1* (E17K) were identified in five EPCs. The tumor suppressor gene *PTEN*, one of the key regulators of the PI3K-AKT-mTOR pathway, had a frameshift mutation (R308 deletion) in one patient resulting in protein inactivation and downstream signal activation. We also detected dual mutations of *PIK3R1* (p85α) (Y165 insertion and G35 deletion) in one patient. *PIK3R1* mutations, located in the SH3 and Rho-GAP domains of p85α, are known to block binding of the PTEN–p85α heterodimer and destabilize PTEN^19^. Activation of MAP3K1 and MAP2K4 represents two contiguous steps within the p38/JNK1 pathway downstream of PI3K-AKT signaling^6,20^. Two EPCs harbored *MAP3K1* truncated mutations, of which one had a concurrent *PIK3CA* (H1047R) mutation and the other had a concurrent *PIK3CA* (E545K) mutation.

As downstream genes of PI3K-AKT signaling, *ULK1* and *RHOA* mutations are rarely detected in breast cancer. Two *ULK1* missense mutations (H72Q and M836I) were predicted as “probably pathogenic” in EPCs. RhoA could repress mTORC1 signaling by reducing the concentration of Rheb·GTP and was a critical downstream target of mTOR involved in actin cytoskeleton remodeling and cell migration [44.45]. One patient harbored an *RHOA* hotspot mutation (G17R) concomitant with *PIK3CA* (E454K).

### Germline and somatic mutations in DNA damage response and repair (DDR) genes

**Fig. 7:**
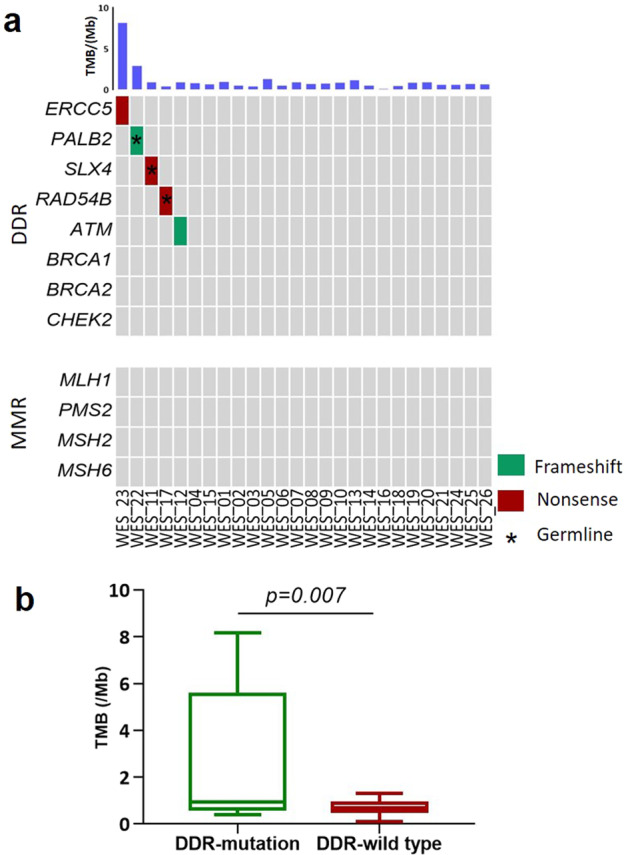
DDR gene mutation in EPC and the TMB comparison between DDR mutated cases and wild type cases. **a** Each column denoted an individual tumor, and each row represented a gene. The different colors represented different mutation types. The TMB was shown at top. **b** The TMB comparison between DDR mutated cases and wild type cases, *p* = 0.007, Unpaired *t* test, Two-Tailed. DDR, DNA damage response and repair; MMR, mismatch repair.

We classified mutations in DDR genes from WES results according to ACMG/AMP classification criteria in DDR genes from WES results^21^. The somatic and germline DDR mutations (“Likely pathogenic” or “Pathogenic”) were listed in Table 1. Five EPC cases harbored DDR gene mutations. All mutation genes were related to homologous recombination repair, including *SLX4*, *ATM*, *RAD54B*, *PALB2*, and *ERCC5* (Fig. 7a). Of the five mutations, three were germline variants and two were somatic. The three germline mutations were classified as “likely pathogenic”, according to ACMG classification criteria. The case with *PALB2* germline mutation has a family history of breast cancer. The detail information about patients with germline DDR gene mutations was listed in the Supplementary Table 4.

**Table 1:** Germline and somatic DDR gene mutations (“Likely pathogenic” or “Pathogenic”) in EPC from WES results.

WES_No.	Gene	Somatic/Germ-line	DNA change	AA change	ACMG Significance	TMB (/Mb)
WES_11	SLX4	Germline	c.5359 C > T	Q1787*	Likely pathogenic	0.93
WES_17	RAD54B	Germline	c.424 G > T	E142*	Likely pathogenic	0.4
WES_22	PALB2	Germline	c.1652dupA	Y551*	Likely pathogenic	2.9
WES_12	ATM	Somatic	c.7723_7729del	P2575fs*	Likely pathogenic	0.9
WES_23	ERCC5	Somatic	c.2527 C > T	Q843*	Likely pathogenic	8.16

*AA* amino acid, *ACMG* the American College of Medical Genetics and Genomics, *DDR* DNA damage response and repair, *TMB* tumor mutation burden, *WES* whole exome sequencing.

*Stop codon.

However, *BRCA1*, *BRCA2*, and mismatch repair (MMR) genes (*MLH1*, *PMS2*, *MSH2*, and *MSH6*) showed neither somatic nor germline mutations in all EPCs. The pathogenic mutation in DDR system may induce a hypermutational phenotype^22^. The average TMB of mutated cases was 2.66 mutations/Mb, which was significantly higher than that of wild type ones (the average TMB: 0.71 mutations/Mb, *p* = 0.007, Unpaired *t* test) (Fig. 7b).

### Comparison of EPCs with luminal A IDC-NSTs in molecular genetic features

We compared the molecular features of EPCs with those of luminal A IDC-NST (*n* = 223, data from The Cancer Genome Atlas, TCGA).

No difference was identified between the two subtypes regarding the TMB (the average TMB of 1.09 [range 0.1–8.17] and 1.02 [range 0.13–6.77] mutations/Mb for EPCs and luminal A IDC-NSTs, respectively; *p* = 0.99, Unpaired *t* test, Two-Tailed) (Supplementary Fig. 2). The predominant mutation spectra of luminal A IDC-NSTs were C > T/G > A and T > C/A > G transitions and C > A/G > T transversions, which were also consistent with that of EPCs.

Moreover, we compared the recurrently mutated genes between our EPCs and luminal A IDC-NSTs (Table 2). Abnormalities of the PI3K-AKT-mTOR pathway were prominent in both of subtypes, with *PIK3CA* being the most commonly mutated gene (46.3% vs. 47.1%, *p* = 1.000, Chi-square test). Some unique features were observed in our EPC. As for chromatin modifications genes, a significantly higher mutation frequency of *ZFPM1* (21.9% vs. 0.0%; *p* < 0.001, Fisher’s exact test) and *CTCF* (12.2% vs. 4%; *p* = 0.048, Chi-square test) were found in EPCs compared with that in luminal A IDC-NSTs. Among PI3K-AKT-mTOR pathway genes*, AKT1* hotspot mutation was significantly more frequent in EPCs (12.2% vs. 3.1%; *p* = 0.024, Chi-square test). Importantly, *TP53* somatic mutations were found in none of our EPCs, whereas it was detected in a considerable proportion of luminal A IDC-NSTs (0.0% vs. 12.1%; *p* = 0.011).

**Table 2:** Comparison the molecular features between EPCs and luminal A IDC-NSTs.

		EPC (*n* = 41)	Luminal A IDC-NST (*n* = 223)	*P* value
Mutation load (mutations/Mb)		1.09	1.02	0.992^a^
PI3K-AKT-mTOR pathway	PIK3CA	19 (46.3%)	105 (47.1%)	1.000
	AKT1	5 (12.2%)	7 (3.1%)	0.024
	ULK1	2 (4.8%)	1 (0.4%)	0.064
	MAP3K1	2 (4.8%)	30 (13.3%)	0.190
	MAP2K4	1 (2.4%)	15 (6.7%)	0.480
	PIK3R1	1 (2.4%)	4 (1.8%)	0.573
	RHOA	1 (2.4%)	0 (0.0%)	0.155
	PTEN	1 (2.4%)	9 (4.0%)	1.000
Chromatin Modification	ZFPM1	9 (21.9%)	0 (0.0%)	0.000
	GATA3	8 (19.5%)	32 (14.2%)	0.476
	CTCF	5 (12.2%)	9 (4.0%)	0.048
	KMT2C	5 (12.2%)	20 (9.0%)	0.560
CGC genes	SPEN	2 (4.8%)	8 (3.6%)	0.657
	CBFB	2 (4.8%)	6 (2.7%)	0.360
	ATM	1 (2.4%)	4 (1.8%)	0.573
	TP53	0 (0.0%)	27 (12.1%)	0.011
Other recurrent mutated genes	ANKRD53	2 (4.8%)	3 (1.3%)	0.173
	RYR3	2 (4.8%)	9 (4.0%)	0.682
	NAALADL2	2 (4.8%)	0 (0.0%)	0.024
	TMED8	2 (4.8%)	0 (0.0%)	0.024
	ABCB8	2 (4.8%)	0 (0.0%)	0.024

*CGC* Cancer Gene Census, *IDC-NST* invasive ductal carcinomas of no special type.

^a^Two-tailed Unpaired *t* test; the remaining used two-tailed Chi square test or Fisher’s exact test.

## Discussion

In this study, a comprehensive genomic landscape of EPCs was generated by WES and TS analysis. We observed that PI3K-AKT-mTOR genes and chromatin modification genes were frequently mutated in EPC. Most importantly, recurrent somatic *ZFPM1* mutations were a characteristic of EPC and concurrent with mutations in the PI3K-AKT-mTOR pathway.

Chromatin modification genes are responsible for various epigenetic regulations that engage gene expression programs. In recent studies, more evidence has demonstrated that inappropriate epigenetic remodeling can also drive tumorigenesis and that chromatin remodeling plays a pivotal role in the early stages of breast pathogenesis^23,24^. Our study found that recurrent mutations in chromatin modification genes (*ZFPM1*, *GATA3*, *CTCF*, and *KMT2C*) were detected in 51.2% of EPCs. Of the chromatin modification genes, *ZFPM1* gene was found to be the most frequently mutated in EPCs, and the mutation of *ZFPM1* gene had not been reported in other breast cancer studies. Moreover, all *ZFPM1* mutations in EPC were frameshift or stop-gain mutations and were thereby presumed to cause loss of the protein function. We inferred that *ZFPM1* might play a role as a potential tumor suppression gene in EPC, which should be further validated by copy number analysis in the region of *ZFPM1* and functional assays.

ZFPM1 protein has been demonstrated to be an important cofactor for GATA proteins in multiple tissues. According to previous studies, the zinc-fingers (ZincF-1, 5, 6, and 9) of ZFPM1 protein are able to directly interact with the N-terminal ZincF of GATA3^15–18^. All *ZFPM1* mutations in our EPC cases were predicted to fail in encoding natural zinc-finger motifs, and thus might impair the interaction with GATA3. On the other hand, all mutated ZFPM1 proteins are predicted to be deleterious in the CTBP-binding region (aa794-800, between ZincF-6 and 7). This region of ZFPM1 protein is able to interact with CTBP protein (C-terminal binding protein, such as CTBP1 and CTBP2) that could promote scaffold interactions between GATA transcriptional factors and chromatin-modifying complexes^25^. GATA3 transcriptional factor relies on the actions of its partner protein to effect changes in the chromatin structure and thereby modulate gene expression^16,17,26^. The effects and mechanisms of *ZFPM1* mutations on GATA transcriptional factors interaction and downstream biological functions in EPCs in EPCs should be further clarified. In addition, *ZFPM1* belongs to the positive regulatory domain (PRDM) gene family, which are involved in human cancer through modulation of several processes, such as epigenetic modifications and genetic reprogramming^27^. Functional studies on epigenetic modifications and genetic reprogramming should be further explored to clarify the potential role of *ZFPM1* mutation in EPC.

As the partner of ZFPM1 protein, GATA3 protein is involved in growth control and the maintenance of luminal epithelial differentiation in mammary tissue. *GATA3* mutations were detected in 19.5% of EPCs. *GATA3* mutations had been reported to contribute to aberrant transactivation activity and tumorigenesis in ER-positive breast cancers^28–30^. Interestingly, our study found that mutations in *ZFPM1* and *GATA3* seemed to show a mutually exclusive pattern in EPCs and further indicated that mutations in *ZFPM1* and *GATA3* might exert common or redundant functional effects.

Apart from chromatin modification genes, we observed an enrichment of alterations in the PI3K-AKT-mTOR pathway in EPC. The frequency of mutations of *PIK3CA* and *AKT1* in EPCs is consistent with previous reports in breast papillary neoplasms^31,32^. Either elevated PI3K activity as a result of *PIK3CA* mutations or downstream AKT activation could cause an oncogenic transformation in mammary epithelial cells and the formation of heterogeneous mammary tumors in vivo^33–35^. As downstream genes of PI3K-AKT signaling, *ULK1* gene could encode an autophagy initiator protein, and its mutation was detected in 4.8% of our EPCs. *ULK1* mutations reduce autophagy-dependent apoptosis directly and induce tumor proliferation and survival by regulating the mTOR-ULK1 signaling axis^36–40^. Given the concurrent *ZFPM1* and PI3K-AKT-mTOR pathway mutations, they might synergistically contribute to the oncogenesis of EPC, and further in vitro functional studies are required.

Given that the majority of our EPCs exhibited luminal A-like phenotype (high hormone receptor expression and low Ki-67 index), we made a comprehensive comparison of molecular genetic features between EPC and luminal A IDC-NSTs for the first time. The somatic mutation load and spectra in our EPCs was similar to luminal A IDC-NSTs. Moreover, frequent mutations in PI3K-AKT-mTOR pathway were detected in both luminal A IDC-NSTs and our EPC cases, and approximately half cases harbored *PIK3CA* mutations. Albeit sharing remarkably similar mutational features with luminal A IDC-NSTs, EPC showed distinctive genetic features. *ZFPM1* mutations were exclusively found in about one-fifth of EPCs, and another chromatin modification gene *CTCF* was also more frequently mutated in EPCs. Among PI3K-AKT-mTOR mutations, *AKT1* activation mutations in our EPCs were also significantly more common than those in luminal A IDC-NSTs, with all mutated EPCs harboring the same mutation of E17K. In addition, EPCs harbored a significantly lower *TP53* mutations. These distinctive genetic features of EPC might underlie its special histological structures.

In our cohort, there was no local recurrence, distant metastasis, or cancer-related death during the follow-up period, consistent with the excellent prognosis of EPCs as previously reported^1,41^. The inactivation of tumor suppression gene *TP53* and DDR genes has been associated with poor prognosis and aggressive clinicopathological features, such as distance metastasis^22,42,43^. No *TP53* gene mutation and infrequent DDR gene mutations were detected in our EPC cases. Our results indicated that the excellent prognosis and indolent biological behavior of EPC might in part be attributed to the intact TP53 and DDR pathways.

Our study had some limitations. First, although our study constitutes the largest series of EPCs with NGS data to date, our sample size is limited owing to the rarity of EPCs. Therefore, the frequency of *ZFPM1* mutation and DDR germline mutation in the EPCs needs more accurate evaluation in large cohorts. Second, only FFPE tumor tissues were available and WES and targeted region sequencing rather than WGS were conducted in our study. The FFPE mutation signature results should be interpreted with caution, and other alteration in EPC, such as structure variation and noncoding region variation, couldn’t been explored in our study. Third, given that the invasive component in our series was small and might be insufficient for DNA sequencing, we did not micro-dissected the invasive component from the EPC component for further comparison in this study. More EPC cases with sufficient invasive component will be collected for comparing the genetic alterations between EPC and its invasive component in our further study.

In conclusion, we revealed for the first time that somatic *ZFPM1* mutations were commonly present and characteristic of EPCs. Moreover, *ZFPM1* mutations co-occurred with mutations in the PI3K-AKT-mTOR pathway and tended to be mutually exclusive with *GATA3* mutations. Our results clarify the genetic mechanism underlying EPC, shed light on EPC tumorigenesis, and may assist diagnosis and treatment for EPC. Our results also suggest that EPC has an excellent prognosis, which might be attributed to the intact TP53 and DDR pathways.

## Methods

### Clinical information and diagnostic criteria for samples

EPCs diagnosed from 2015 to 2018 were retrospectively retrieved from the surgical pathology files of Peking Union Medical College Hospital. The histological slides were reevaluated by three experienced pathologists (Z.L., H.W., and X.L.) to verify the diagnosis according to the 2019 WHO classification of tumors of the breast^44^. The exclusion criteria were as follows: (i) patients with multiple tumors; (ii) patients for which the area of accompanied invasive carcinoma was larger than the area of EPC; and (iii) patients who did not have paired blood/normal tissue. A total of 41 patients were selected for this study. All tumor samples were obtained from surgical resection and fixed in formalin and embedded in paraffin. The study protocol was approved by the Ethical Committee of Peking Union Medical University Hospital. Our study is compliant with the “Guidance of the Ministry of Science and Technology (MOST) for the Review and Approval of Human Genetic Resources”. We got the formal approval for the export of human genetic material or data. The approval ID is 2021BAT1149. The clinicopathological information was listed in Supplementary Data 1.

### DNA extraction

For FFPE samples, 5 mm sections were cut, placed onto glass slides, and stained with hematoxylin and eosin (H&E). Tumor samples were screened to ensure the percentage of tumor cells was greater than 80%. DNA extraction from FFPE tissues was performed using the QIAmap DNA FFPE Tissue Kit (Qiagen, 56404). The tumor cells of EPCs were enriched using an H&E-stained section as a template, and tumor areas (only the EPC areas) were dissected using a clean scalpel blade. For whole blood samples, genomic DNA was extracted by the DNeasy Blood & Tissue Kit (Qiagen). The quantity of DNA was analyzed by Qubit (Invitrogen, Qubit2.0), and DNA quality was determined using PicoGreen™ fluorometric analysis as well as visual inspection of agarose gel electrophoresis images. The sample tissue information was shown in Supplementary Table 1.

### Whole-exome sequencing and mutation calling

Whole-exome sequencing was performed for 26 patients with matched tumor and normal samples. Sequencing libraries were generated using the Agilent Sure Select Human All Exon V6 Kit (Agilent Technologies, CA, USA) following the manufacturer’s recommendations, and index codes were added to each sample. Tumor/normal paired DNA libraries were sequenced on an Illumina HiSeq X Ten platform, and 150 bp paired-end reads were generated. To minimize the inclusion of sequencing artefacts related to formalin fixation, bioinformatics analysis included quality control, read mapping to the reference sequence, variant calling, identification of candidate somatic variants, and functional annotation. The quality control was conducted: (1) remove reads with sequencing adapter; (2) remove reads with a ratio of N base (N represents indeterminable base information) greater than 10%; (3) the sequencing error rate and percentage of reads with Q30 (the percent of bases with Phred-scaled quality scores greater than 30) were calculated and summarized, and to remove low quality reads (the ratio of base with Q phred≤20 greater than 50% in the read). The sequencing reads were aligned to the reference genome (hg19) using bwa and samblaster, and soft-clipped reads were filtered. Somatic mutation analysis was performed with Mutect. To filter SNVs, filtering parameters including coverage (>10X), variant frequency (>0.08), variant read support (>3 reads) were applied. The pileup files created by SAMtools mpileup for tumor and matched normal samples. Then, the C > T or G > A mutation sites were filtered by the strand bias: (1) Discarded mutant sites that occurred only in plus or minus strand; (2) Reserved mutant sites that the ratios of the number of sites occurred in plus or minus strand within 0.2–5. To make the results more reliable, we also adopted a relative deep sequencing depth. All candidate variants were visually inspected in Integrated Genome Viewer (IGV) (Supplementary Data 2). Tumor mutation burden (TMB) was defined as the number of detected mutations over the region of a tumor genome. TMB was calculated for using coding base substitutions and indel alterations. Rearrangements, fusions, and copy number variants were excluded.

### Mutation spectrum and mutation signature analysis

Mutation spectrum and signature analysis were performed in each WES tumor sample. Mutation spectrum bar plot was drawn to present mutation spectrum of each sample. By nonnegative matrix factorization (NMF), we conducted cluster analyses on 96 somatic mutation types and acquired three different mutation signatures. Then, mutation spectra were clustered with 30 known signatures on COSMIC to explain mutation process of samples. Signatures A, B, and C were identified in tumor samples and the distribution of them was presented. The cosine similarity (α) has a value between 0 and 1. Two mutational profiles are identical when the cosine similarity is 1, and independent when the cosine similarity is 0.

### Targeted sequencing

To validate the WES results, deep targeted sequencing was conducted. The 26 paired WES samples were then subjected to TS analyses. Moreover, to expand the sample size, 15 additional paired EPC samples were submitted for TS. Finally, sequencing libraries were prepared from 41 patients.

Sequencing probes were designed on the Agilent Technologies website for 185 genes. The panel genes included genes frequently mutated in EPCs, genes likely to be pathogenic in EPCs, genes mutated in intraductal papillary neoplasms, and genes associated with invasive breast carcinoma^5,6,45^. The panel genes are listed in Supplementary Table 2. Target-enriched libraries were then sequenced on the Illumina HiSeq X Ten platform, and 150 bp paired-end reads were generated. For paired tumor and normal samples, the analysis was performed using the same methods as the exome sequencing analysis. When variant allele frequencies were greater than 8%, MuTect and Strelka were used for calling somatic single nucleotide variants (SNVs) and small indels, respectively. All candidate variants were visually inspected in IGV (Supplementary Data 3). A KEGG pathway and GO analysis was performed based on genes affected by nonsynonymous pathogenic somatic mutations in EPCs (*n* = 41), and pathways found to be significantly enriched (*p* < 0.01) were selected^46,47^.

### Sanger sequencing

Sanger sequencing was conducted to validate recurrently mutated genes. Primer information for the Sanger sequencing experiments was provided in Supplementary Table 3. PCR amplification was conducted with Phusion Polymerase (Life Technologies). The PCR program included one cycle at 95 °C for 10 min; 35 cycles at 95 °C for 30 s, 62 °C for 30 s and 72 °C for 1 min; and one cycle at 72 °C for 5 min. Bidirectional sequencing of the generated PCR amplicons was performed using the BigDye Terminator v.3.1 kit (Applied Biosystems, Thermo Fisher Scientific, USA). Products were analyzed by the ABI PRISM 3730 Genetic Analyzer (Applied Biosystems, Thermo Fisher Scientific, USA).

### Determination of significantly mutated genes and mutation landscape

Significantly mutated genes were defined using MutSigCV^48^. Cancer Gene Census (CGC513) databases were utilized to characterize the genetic landscape^49^. The reads with variant allele frequencies (VAFs) lower than 5% were removed from the raw data.

### Comparative analysis with luminal A invasive ductal carcinomas of no special type (IDC-NST)

The comparison between EPC and luminal A IDC-NSTs in molecular features were conducted. Molecular data for luminal A IDC-NSTs from The Cancer Genome Atlas (TCGA; *n* = 223) were obtained from the TCGA Data Portal (https://tcga-data.nci.nih.gov/docs/publications/brca_2012/)^6^. The mutation spectrum analysis in luminal A IDC-NST was performed as described for EPC.

### Statistical analysis

Concurrent mutation and mutual exclusivity were tested using Chi-square test or Fisher’s exact test. Comparisons of continuous and categorical features were performed using Unpaired *t* test and Fisher exact tests as appropriate. All *p* values were two-tailed, and 95% confidence intervals were adopted for all analyses. Statistical analyses were performed with R v3.1.2 and SPSS v24.

### Reporting summary

Further information on research design is available in the Nature Research Reporting Summary linked to this article.

## Supplementary information

Supplementary Information

Supplementary Data 1

Supplementary Data 2

Supplementary Data 3

Supplementary Data 4

Reporting Summary

## Data Availability

The data generated and analysed during this study are described in the following data record: 10.6084/m9.figshare.14410811^50^. The raw sequencing data are openly available in the NCBI Sequence Read Archive, and can be accessed via the following BioProject accession: https://identifiers.org/bioproject:PRJNA685582^51^. These data have also been deposited in the Genome Sequence Archive (Genomics, Proteomics & Bioinformatics 2017) in the National Genomics Data Center (Nucleic Acids Res 2020), Beijing Institute of Genomics (China National Center for Bioinformation), Chinese Academy of Sciences. The accession code is HRA000480. The TCGA dataset used in the related article is from https://tcga-data.nci.nih.gov/docs/publications/brca_2012/. Additional data underlying the figures of the related article are openly available in the files included with the figshare data record.
