# Supplementary material for: Next-generation sequencing revealed recurrent ZFPM1 mutations in encapsulated papillary carcinoma of the breast
Source: NPJ Precis Oncol. 2021 May 18;5:42. doi: 10.1038/s41698-021-00180-5 (PMC8131604; doi:10.1038/s41698-021-00180-5)
Supplement: Supplementary file 1 — Supplementary Information [file 41698_2021_180_MOESM1_ESM.pdf]

Supplementary Information

Next-generation sequencing revealed recurrent ZFPM1 mutations in encapsulated papillary carcinoma of the breast

Supplementary Figure 1..**Page 2.**
Supplementary Figure 2..**Page 4.**
Supplementary Figure 3..**Page 5.**
Supplementary Figure 4..**Page 6.**
Supplementary Table 1..**Page 7.**
Supplementary Table 2..**Page 9.**
Supplementary Table 3..**Page 10.**
Supplementary Table 4..**Page 13.**

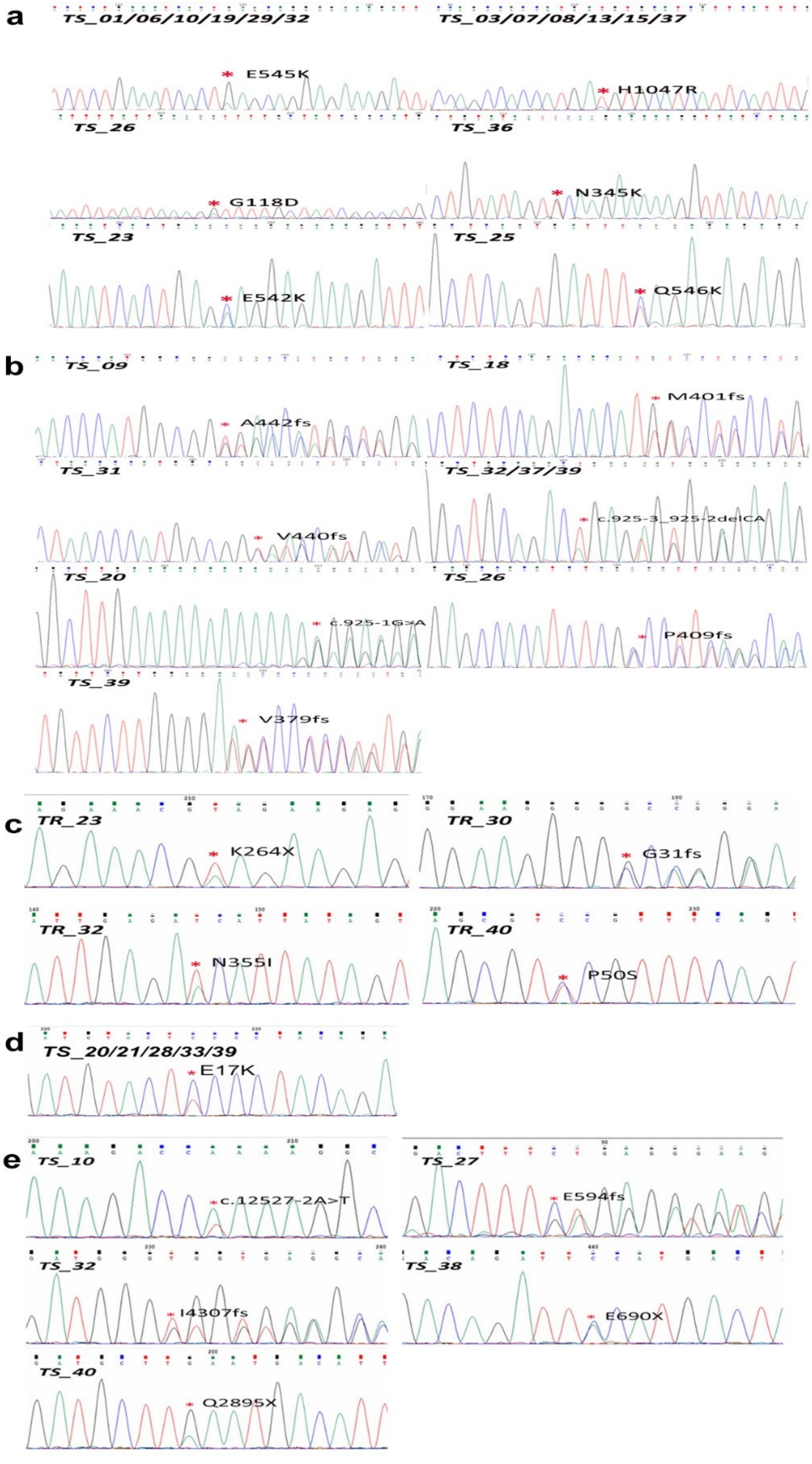
a
TS_01/06/10/19/29/32
E545K
TS_03/07/08/13/15/37
H1047R
TS_26
G118D
TS_36
N345K
TS_23
E542K
TS_25
Q546K
b
TS_09
A442fs
TS_18
M401fs
TS_31
V440fs
TS_32/37/39
c.925-3_925-2delCA
TS_20
c.925-1G>A
TS_26
P409fs
TS_39
V379fs
c
TR_23
K264X
TR_30
G31fs
TR_32
N355I
TR_40
P50S
d
TS_20/21/28/33/39
E17K
e
TS_10
c.12527-2A>T
TS_27
E594fs
TS_32
I4307fs
TS_38
E690X
TS_40
Q2895X

Supplementary Fig. 1. Some recurrent mutated genes for Sanger sequencing. (a) *PIK3CA* mutations, **(b)** *GATA3* mutations, **(c)** *CTCF* mutations, **(d)** *AKT1* mutations, **(e)** *KMT2C* mutations.

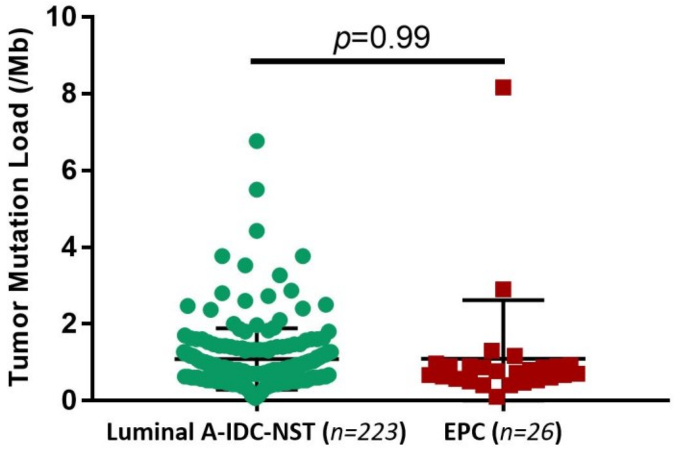

Supplementary Fig. 2. Mutation load in EPCs and luminal A IDC-NST. Two-tailed Unpaired t test.

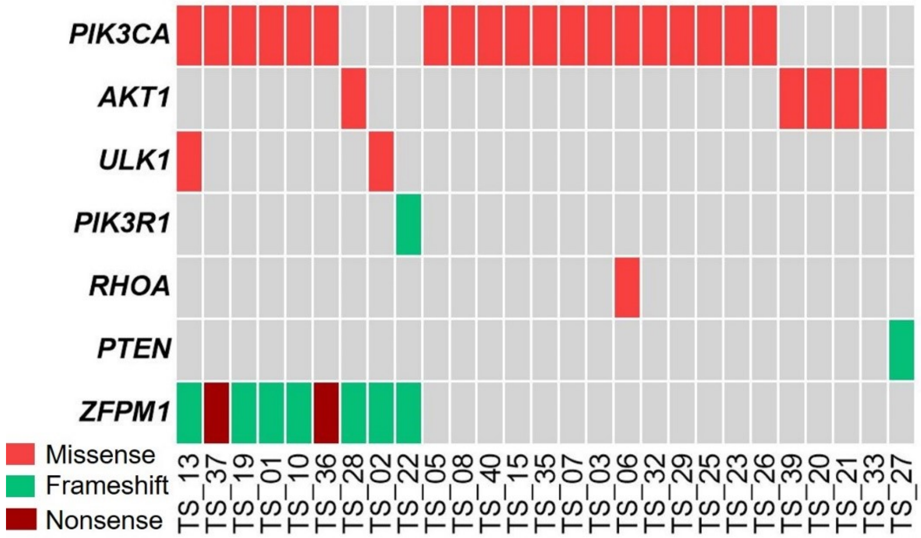

Supplementary Fig. 3 Co-occurrence of *ZFPM1* and PI3K-AKT-mTOR pathway mutations. Twenty-seven samples had PI3K-AKT-mTOR pathway mutations, and nine samples had *ZFPM1* mutations, p=0.017, Fisher's exact test. Each column denotes an individual tumor, and each row represents a mutation. The different colors represented different mutation types.

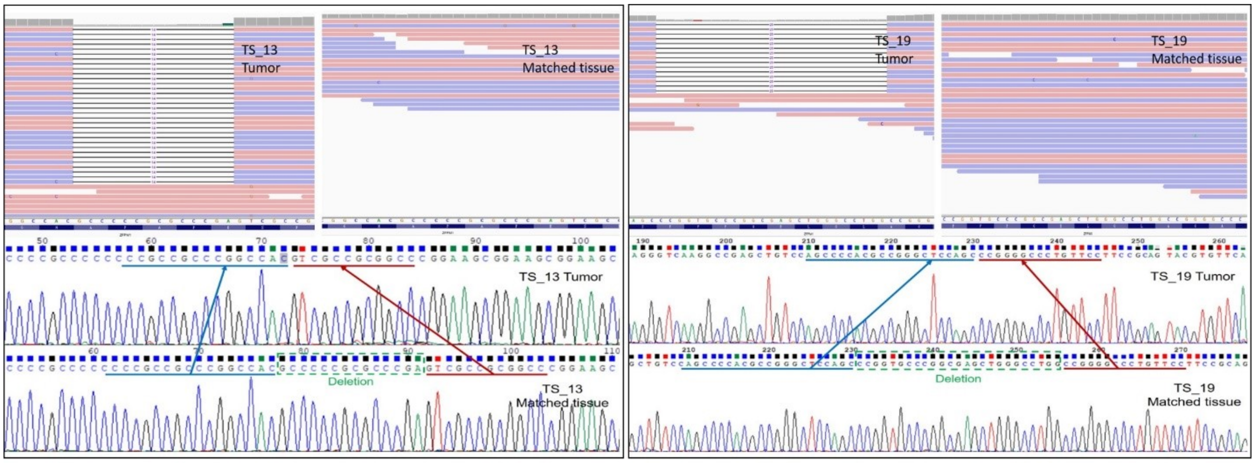

A B

Supplementary Figure 4. ZFPM1 mutation in TS_13 (A) and TS_19 (B) revealed by NGS and Sanger sequencing. High variant allele frequency (VAF) of ZFPM1 mutation were revealed by NGS for TS_13 and TS_19, and the chromatographic peak corresponding to ZFPM1 mutation was shown at the same position.

Supplementary Table 1. The sample tissue information

	Targeted sequencing			Whole-exome sequencing		
Patient No.	TS_ No.	Tumor	Normal	WES_ No.	Tumor	Normal
Patient 01	TS__01	FFPE	Whole blood	WES_ 01	FFPE	Whole blood
Patient 02	TS_ 02	FFPE	Whole blood	WES_ 02	FFPE	Whole blood
Patient 03	TS_ 03	FFPE	Whole blood	WES_ 03	FFPE	Whole blood
Patient 04	TS_ 04	FFPE	Whole blood	WES_ 04	FFPE	Whole blood
Patient 05	TS_ 05	FFPE	Whole blood	WES_ 05	FFPE	Whole blood
Patient 06	TS_ 06	FFPE	Whole blood	WES_ 06	FFPE	Whole blood
Patient 07	TS_ 07	FFPE	Whole blood	WES_ 07	FFPE	Whole blood
Patient 08	TS_ 08	FFPE	Whole blood	WES_ 08	FFPE	Whole blood
Patient 09	TS_ 09	FFPE	Whole blood	WES_ 09	FFPE	Whole blood
Patient 10	TS_ 10	FFPE	Whole blood	WES_ 10	FFPE	Whole blood
Patient 11	TS_ 11	FFPE	Whole blood	WES_ 11	FFPE	Whole blood
Patient 12	TS_ 12	FFPE	Whole blood	WES_ 12	FFPE	Whole blood
Patient 13	TS_ 13	FFPE	Whole blood	WES_ 13	FFPE	Whole blood
Patient 14	TS_ 14	FFPE	Whole blood	WES_ 14	FFPE	Whole blood
Patient 15	TS_ 15	FFPE	Whole blood	WES_ 15	FFPE	Whole blood
Patient 16	TS_ 16	FFPE	Whole blood	WES_ 16	FFPE	Whole blood
Patient 17	TS_ 17	FFPE	FFPE lymph node tissue	WES_ 17	FFPE	FFPE lymph node tissue
Patient 18	TS_ 18	FFPE	FFPE lymph node tissue	WES_ 18	FFPE	FFPE lymph node tissue
Patient 19	TS_ 19	FFPE	FFPE lymph node tissue	WES_ 19	FFPE	FFPE lymph node tissue
Patient 20	TS_ 20	FFPE	FFPE lymph node tissue	WES_ 20	FFPE	FFPE lymph node tissue
Patient 21	TS_ 21	FFPE	FFPE lymph node tissue	WES_ 21	FFPE	FFPE lymph node tissue
Patient 22	TS_ 22	FFPE	FFPE lymph node tissue	WES_ 22	FFPE	FFPE lymph node tissue
Patient 23	TS_ 23	FFPE	FFPE lymph node tissue	WES_ 23	FFPE	FFPE lymph node tissue
Patient 24	TS_ 24	FFPE	FFPE lymph node tissue	WES_ 24	FFPE	FFPE lymph node tissue
Patient 25	TS_ 25	FFPE	FFPE lymph node tissue	WES_ 25	FFPE	FFPE lymph node tissue
Patient 26	TS_ 26	FFPE	FFPE lymph node tissue	WES_ 26	FFPE	FFPE lymph node tissue
Patient 27	TS_ 27	FFPE	FFPE lymph node tissue			
Patient 28	TS_ 28	FFPE	FFPE lymph node tissue			
Patient 29	TS_ 29	FFPE	FFPE lymph node tissue			
Patient 30	TS_ 30	FFPE	FFPE lymph node tissue			
Patient 31	TS_ 31	FFPE	FFPE lymph node tissue			
Patient 32	TS_ 32	FFPE	FFPE lymph node tissue			
Patient 33	TS_ 33	FFPE	FFPE lymph node tissue			
Patient 34	TS_ 34	FFPE	FFPE lymph node tissue			
Patient 35	TS_ 35	FFPE	FFPE lymph node tissue			

Patient 36	TS_ 36	FFPE	FFPE lymph node tissue			
Patient 37	TS_ 37	FFPE	FFPE lymph node tissue			
Patient 38	TS_ 38	FFPE	FFPE lymph node tissue			
Patient 39	TS_ 39	FFPE	FFPE lymph node tissue			
Patient 40	TS_ 40	FFPE	FFPE lymph node tissue			
Patient 41	TS_ 41	FFPE	FFPE lymph node tissue			

Supplementary Table 2. Panel gene list

ABCB8	*CD84*	*GRID2*	*MSI2*	*PCDHA5*	*SCAF4*	*ZAK*
ADK	*CDH1*	*GTF3C2*	*MTSS1*	*PCDHA9*	*SDC4*	*ZBED1*
AGAP4	*CDH3*	*HECTD1*	*MUC2*	*PCDHGA10*	*SEC14L5*	*ZC3HAV1*
AKAP9	*CDKN1B*	*HEMK1*	*MYC*	*PHKA2*	*SETD2*	*ZCCHC16*
AKT1	*CDKN2A*	*HERC1*	*MYH4*	*PIK3CA*	*SLC8A2*	*ZFPM1*
AKT2	*CLTC*	*HMCN1*	*MYO1D*	*PIK3R1*	*SLIRP*	*ZNF608*
ANKRD53	*CNTN4*	*HRAS*	*MYOM3*	*PLD2*	*SMG6*	
APOA4	*CROCC*	*HYDIN*	*NAALADL2*	*PLEKHG5*	*SP1*	
ARID1A	*CSMD3*	*IDH2*	*NAP1L2*	*POTEH*	*SPEN*	
ARMCX6	*CTCF*	*IGFN1*	*NAT2*	*PPARGC1B*	*SVEP1*	
ARNTL2	*CTNNA2*	*ITGA8*	*NBPF10*	*PPEF2*	*TBC1D3F*	
ATM	*CTPS2*	*ITGB4*	*NBPF8*	*PPP2R1B*	*TBP*	
ATP13A1	*CUX1*	*JAK2*	*NCAN*	*PRAMEF1*	*TBX1*	
AXDND1	*CXCR6*	*KAT2A*	*NCOR1*	*PRKCA*	*TCF21*	
BCL6	*DEPDC1B*	*KCNV1*	*NCOR2*	*PRKCZ*	*TECTA*	
BCORL1	*DGKG*	*KDM4C*	*NFKBIE*	*PRKG1*	*TET2*	
BRAF	*DMXL1*	*KIF1C*	*NLRP3*	*PRPF8*	*TFAP2B*	
BRCA1	*DNAH1*	*KIF20B*	*NOTCH1*	*PSD2*	*THRAP3*	
BRCA2	*DNAJC1*	*KMT2C*	*NOTCH2*	*PTEN*	*TMED8*	
BSN	*DNPEP*	*KRT5*	*NR3C1*	*PTH1R*	*TMEM86B*	
BUD13	*DOPEY2*	*KY*	*NRAS*	*PTPN12*	*TP53*	
C10orf76	*DRP2*	*LAMA5*	*NRG1*	*PXDNL*	*TSHZ3*	
C3	*ELAVL3*	*LOH12CR1*	*NUTM2B*	*RASA3*	*TTN*	
C8B	*ENTPD1*	*MACF1*	*ODF3L2*	*RB1*	*TUBA1A*	
CACNA1E	*EPSTI1*	*MAGEC1*	*OGFR*	*RHOA*	*U2AF1*	
CBFB	*ERBB2*	*MAOB*	*OMD*	*ROBO2*	*ULK1*	
CBL	*FER1L5*	*MAP2K4*	*OR2T2*	*RPL10*	*UNC80*	
CCDC33	*FGFR1*	*MAP3K1*	*OR4D5*	*RYR3*	*USF1*	
CCDC38	*GATA3*	*MAPKAPK3*	*OR5W2*	*SAMD4A*	*USP26*	
	GPR32	*MAST2*	*PAN2*	*SARNP*	*VCL*	

Supplementary Table 3. The primers information for sanger sequencing

	Location	Forward PRIMER	Reverse PRIMER	Length (bp)	
SPEN	chr1-16254852	AGGGCATCCTACGACTATAACC	TCCAGTTTCTCATATCTTGGAG	473	NM_015001::c.G2117A:p.R706H
SPEN	chr1-16256190	TTGCCAGTATTTCTGTTGGGTC	GTCATCAGTGACATCTTGAGGG	458	NM_015001:c.C3455A:p.S1152X
IGFN1	chr1-201180874	GATGGTTTAGGGGGTTCTGAAG	TCCCTGAACCATCTCCATAACC	509	NM_001164586:c.G6853A:p.G2285S
NAALADL2	chr3-175181225	TCATCACTGCGTAACAAACACC	AGGAAAGAGAAGGAAGACCCAC	471	NM_207015:c.C1271G:p.T424R
PIK3CA	chr3-178917478	TTGCTGCCTTTGCTCTAAATTG	AGACACAGGTAGAAGACTGCAC	500	NM_00621:c.G353A:p.G118D
PIK3CA	chr3-178921553	GAAAAACCTTACAGGAAATGGC	ATTAACAAAGGACTGTGAGCTG	495	NM_006218:c.T1035A:p.N345K
PIK3CA	chr3-178936082-91-94	CTTAGATTGGTTCTTTCCTGTC	TGCCAACTACCAATGTAGTATG	518	NM_006218:c.G1624A:p.E542K<br>NM_006218:c.G1633A:p.E545K<br>NM_006218c.C1636A:p.Q546K
PIK3CA	chr3-178952085	ACATCATTTGCTCCAAACTGAC	AGTGCTATCAAACCCTGTTTGC	503	NM_006218:c.A3140T:p.H1047L/R
SETD2	chr3-47163187	TCATCCGAATCTGTATCTTCTG	CATGCAGGGAAAGAAACAATAG	407	NM_014159::c.C2939G:p.S980C
TET2	chr4-106157007	CAACACAGCACTATCTGAAACC	GTTTCAACACTGGGGACATAAG	438	NM_001127208:c.1909delG:p.E637fs
DEPDC1B	chr5-59982904	ACCCACTGTTCAGTGAACAAGG	CTCATTCCACTAGGGGAAAGAA	395	NM_001145208:c.G199C:p.V67L
PIK3R1	chr5-67522605	CAGTCACCTCTCCTCTTAAACC	TGACCTTGTTGTTCAACATCTG	502	NM_181523:c.103_104del:p.G35fs
PIK3R1	chr5-67590990	AAATGACAGGAAGAGAAGCCAC	TTCAGTGTTTTCATTGCCCAAC	470	NM_001242466:c.495dupT:p.Y165fs
ABCB8	chr7-150728325	AAGCATTTCCATTTTGTGTGGC	ATGGCCTTTCACATCCCTAGAC	475	NM_001282291:c.96-3C>T
KMT2C	chr7-151846093	GATCAGGTTTAAGGGAAGTGCC	TTCAGTCATACTGCTCAGCTTC	500	NM_170606:c.12918dupC:p.I4307fs
KMT2C	chr7-151848668	CCATAAGTGGTGTCTCTCTCTG	CCATTGCTTTCCCAGCTATTTG	463	NM_170606:c.12527-2A>T

KMT2C	chr7-151873855	ATGGCATTATCCAAAACACGG	TCACCACAGAAAAAATCCAC	476	NM_170606:c.C8683T:p.Q2895X
KMT2C	chr7-151945451	TGACTCTGTCTCAGATGATAAC	AGTATCATCCCAACATACAGTG	493	NM_170606:c.G2068T:p.E690X
KMT2C	chr7-151946992	ACAGCAATCGCAAAAATGTC	CATAAAAAGCCACTGAGATG	476	NM_170606:c.1780_1781del:p.E594fs
GATA3	chr10-8111432-5	TTACTGCAATCCTGACATGCTC	TATTTGGAACCTGTCATCTGCC	465	NM_001002295:c.925-1G>A<br>NM_001002295:c.925-3_925-2delCA
GATA3	chr10-8115786-852-874	TGTTTAGATTAACAGACCCCTG	GGAACACAGACACCACAGTGAG	468	NM_001002295:c.1136_1151del:p.V379fs<br>NM_001002295:c.1202dupT:p.M401fs<br>NM_001002295:c.1224dupG:p.S408fs
GATA3	chr10-8115968-72	GGATTTGCTAGACATTTTTCGG	TTGGAACACAGACACCACAGTG	416	NM_001002295:c.1322dupC:p.T441fs<br>NM_00100229:c.1318_1321del:p.V440fs
PTEN	chr10-89720771	ATTCTTCATACCAGGACCAGAG	CAGCAAACAACGAAGAATTAGG	426	NM_000314:c.923_957del:p.R308fs
ULK1	chr12-132380339	TGCACGTTTGGCTTTCAGAAG	GAAGTCACAGCAAGAGCCAGC	416	NM_003565:c.T216G:p.H72Q
ULK1	chr12-132403223	ACTGGCTCTGCCAGCTCTTC	ACACACCCGACACCCCACACA	365	NM_003565:c.G2508A:p.M836I
AKT1	chr14-105246551	AGCCAGTGCTTGTTGCTTGC	AGCCCGTTTTCAGACACAGC	510	NM_001014431:c.G49A:p.E17K
TMED8	chr14-77808250	TCTTGCCTATCCCAAACAAGAG	CAAAAGATCACCAGACATTTCC	446	NM_213601:c.G842A:p.R281Q
RYR3	chr15-33927862	CAATCCCTCAGTCAGTGGTAGC	TTTGGAGAAGGAAAACTTGGTC	463	NM_001036:c.C3223T:p.R1075W
CBFB	chr16-67063717	CAGCCTCTGCTTGCCCTTATC	ATCCCGAGTAGGAAGTGAGACG	321	NM_001755:c.165+1->T
CTCF	chr16-67644825-923	TGCCTAATTCATTCACCAAAGG	CACTTCATTTTCATAAGCCCCC	536	NM_006565:c.189delG:p.M63fs<br>NM_006565:c.91delG:p.G31fs
CTCF	chr16-67654645	CTATACCCGTATTCATTTACCC	TAAACAATGGGGACACACAGTG	425	NM_001191022:c.C148T:p.P50S
CTCF	chr16-67663373	GAACCCAGCCTTATCCATTTCC	GCCCCAGAAAAACACTAACTGC	473	NM_001191022:c.A790T:p.K264X
CTCF	chr16-67671639	CTCTGGACCGCTATCTAATAAG	AACACAGCCCAGAGAAGTCCTG	355	NM_001191022:c.A1064T:p.N355I

ZFPM1	chr16-88593284-97	GGTCAACTATGGCACAGATGG	CAGTAGAGTGTCTGCCTCATGC	455	NM_153813:c.466_469del:p.L156fs NM_153813:c.466_469del:p.L156fs
ZFPM1	chr16-88600168	TGTTCCTTCCGCAGTACG	CTGAAGCGGATGTTGCAGG	508	NM_153813:c.C1802A:p.S601X NM_153813:c.1822_1828del:p.D608fs
ZFPM1	chr16-88600759	TGCGCACGCGCAGACGCCGCAA	GGAAATGCTCCAGCAGGTCC	408	NM_153813:c.2394dupC:p.S798fs
ZFPM1	chr16-88599076	GACGCCGCAAGCTCTACGA	GAGGCTGTGGAAGCTCACG	263	NM_153813:c.1001_1002insCTGCGAGCGGCAC:p.N334fs
ZFPM1	chr16-88600034	GTTCCTTCCGCAGTACGTGT	AGGTGATCTCGCACTCGAAG	174	NM_153813:c.1669_1673del:p.G557fs
ZFPM1	chr16-88600652	GCGCAGACGCCGCAAGCTCTACGA	CGTGCACTCGTGGTAGTCGGCCAGC	241	NM_153813:c.2287_2300del:p.A763fs
ZFPM1	chr16-88599902	AGGACTGGACAGAAAGGC	GAACACGTACTGCGGAAGG	317	NM_153813:c.1537_1561del:p.P513fs

Supplementary Table 4. The detail information for patients with DDR germline mutations

WES_No.	WES_11	WES_17	WES_22
Gene	*SLX4*	*RAD54B*	*PALB2*
Somatic/Germline	Germline	Germline	Germline
DNA change	c.5359C>T	c.424G>T	c.1652dupA
AA change	Q1787*	E142*	Y551*
Clinvar dataset	No	No	No[1]
ACMG classification	Likely pathogenic	Likely pathogenic	Likely pathogenic
TMB (/Mb)	0.93	0.4	2.9
Age at EPC diagnosis	61	69	78
Family history of breast cancer	No	No	Yes
Relative with breast cancer	No	No	Mother

Abbreviations: AA, amino acid; ACMG, the American College of Medical Genetics and Genomics; DDR, DNA damage response and repair; TMB, tumor mutation burden; WES, whole exome sequencing.

1.The same AA change [NM_024675.3(*PALB2*):c.1653T>A (p. Y551*)] has been previously described in Clinvar as pathogenic/likely pathogenic variant (https://www.ncbi.nlm.nih.gov/clinvar/variation/1243/).
